# GPCR Pharmacological Profiling of Aaptamine from the Philippine Sponge *Stylissa* sp. Extends Its Therapeutic Potential for Noncommunicable Diseases

**DOI:** 10.3390/molecules26185618

**Published:** 2021-09-16

**Authors:** Harmie Luyao, Hendrik Luesch, Mylene Uy

**Affiliations:** 1 Department of Chemistry, Mindanao State University—Iligan Institute of Technology, Iligan City 9200, Philippines; harmie.luyao@g.msuiit.edu.ph; 2 Department of Medicinal Chemistry and Center for Natural Products, Drug Discovery, and Development (CNPD3), University of Florida, Gainesville, FL 32610, USA; 3 Premier Research Institute of Science and Mathematics (PRISM), Mindanao State University—Iligan Institute of Technology, Iligan City 9200, Philippines

**Keywords:** aaptamine, GPCRs, ADRA2C, ADRB2, DRD4, CXCR7, NCDs

## Abstract

We report the first isolation of the alkaloid aaptamine from the Philippine marine sponge *Stylissa* sp. Aaptamine possessed weak antiproliferative activity against HCT116 colon cancer cells and inhibited the proteasome in vitro at 50 µM. These activities may be functionally linked. Due to its known, more potent activity on certain G-protein coupled receptors (GPCRs), including α-adrenergic and δ-opioid receptors, the compound was profiled more broadly at sub-growth inhibitory concentrations against a panel of 168 GPCRs to potentially reveal additional targets and therapeutic opportunities. GPCRs represent the largest class of drug targets. The primary screen at 20 µM using the β-arrestin functional assay identified the antagonist, agonist, and potentiators of agonist activity of aaptamine. Dose-response analysis validated the α-adrenoreceptor antagonist activity of aaptamine (ADRA2C, IC_50_ 11.9 µM) and revealed the even more potent antagonism of the β-adrenoreceptor (ADRB2, IC_50_ 0.20 µM) and dopamine receptor D4 (DRD4, IC_50_ 6.9 µM). Additionally, aaptamine showed agonist activity on selected chemokine receptors, by itself (CXCR7, EC_50_ 6.2 µM; CCR1, EC_50_ 11.8 µM) or as a potentiator of agonist activity (CXCR3, EC_50_ 31.8 µM; CCR3, EC_50_ 16.2 µM). These GPCRs play a critical role in the treatment of cardiovascular disease, diabetes, cancer, and neurological disorders. The results of this study may thus provide novel preventive and therapeutic strategies for noncommunicable diseases (NCDs).

## 1. Introduction

Nakamura et al. first reported the isolation of aaptamine, a benzo [de] (1,6)–naphthyridine alkaloid with α-adrenoreceptor blocking activity, from the marine sponge *Aaptos aaptos* of Okinawa island [1]. Aaptamine was believed to be a taxonomic marker for the marine sponge *Suberitidae*, which belongs to the order *Hadromerida* (sub-class *Tetractinomorpha*) [2]. Later, this claim was rejected when aaptamine was isolated in large quantities from different genera (*Aaptos*, *Suberites*, *Luffariella*, *Xestospongia*, and *Hymeniacidon*). It was proposed that the source of aaptamine within these sponges could be a symbiotic microbe since these sponges are taxonomically unrelated and found in different geographic locations [3].

The compound has previously been shown to possess α-adrenoreceptor-blocking activity [1], antimicrobial, antifungal, antifouling, cytotoxic, antiviral, antioxidant, and cancer-preventive activities [4], as well as proteasome inhibitory activity [5]. In recent studies, aaptamine was reported to have antidepressant-like effects because the compound was determined to be a “balanced” agonist of the delta opioid receptor (δ-OR, EC_50_ 5.1 µM), in addition to being a mu opioid receptor (μ-OR) agonist (EC_50_ 10.1 µM) [6]. Structure-activity relationship (SAR) studies of aaptamine showed that two of its analogs, 9-demethyl aaptamine and demethyl(oxy)-aaptamine, were more potent against δ-OR compared to aaptamine, with EC_50_ values of 4.1 and 2.3 µM, respectively [6]. 

In drug discovery, G-protein coupled receptors (GPCRs) are considered one of the most flourishing therapeutic target families and have experienced a great shift from off-target ligand screening to knowledge-driven drug design [7]. GPCRs (a protein family encoded by the human genome) are found on the cell membrane and convert extracellular signals to key physiological effects [8]. The seven-transmembrane protein endows activation of the GPCR by external signals through coupling to different G-proteins or arrestins. The diversified downstream signaling pathways make GPCRs attractive for drug development [9]. GPCRs have been shown to be involved in many noncommunicable diseases (NCDs), such as type 2 diabetes mellitus (T2DM), heart disease, obesity, depression, cancer, Alzheimer’s disease, and many others [10]. (NCDs are chronic and noncontagious health conditions [11]). There are 826 human GPCRs recorded so far; ~42% of those are regarded as druggable and 20% are validated drug targets [12]. Recent reports indicate that ~700 approved drugs target GPCRs, suggesting that approximately 35% of approved drugs target GPCRs [10] and ~60% of drug candidates currently in clinical trials target GPCRs [13]. GPCRs and GPCR-related proteins upstream or downstream constitute approximately 17% of protein targets for approved drugs (GPCRs alone recorded as ~12%). Drugs that target GPCRs and GPCR-related proteins are usually peptides and small molecules [10].

In this study, aaptamine was obtained from the Philippine marine sponge *Stylissa* sp., which is the first report of this compound in *Stylissa* sp. Since aaptamine is known to modulate selected GPCRs [1,6], we aimed to obtain a more comprehensive GPCR pharmacological profile. Aaptamine was found to antagonize ADRA2C, ADRB2, and DRD4, and to activate CXCR7 β-arrestin signaling. We also explored its function as a potentiator of the agonist activity of other chemokine receptors (CXCR3, CCR3). Indeed, aaptamine may have potential as a starting point for the treatment of NCDs. The α-adrenergic antagonistic activity of the compound was first suggested by Ohizumi et al. who concluded that aaptamine has a cardiac activity [14]. To date, activation of CXCR7 and antagonistic activity of aaptamine against ADRB2 and DRD4 have not been reported. Hence, the findings gathered in this study are a valuable addition to the reported activities of aaptamine in the literature. 

## 2. Results and Discussion

### 2.1. Collection, Isolation, and Structure Elucidation

*Stylissa* sp. was collected off the coast of Lipata, Surigao City, Mindanao, Philippines at a depth of 5–10 m. The sponge extract (50% methanol and 50% ethyl acetate) was sequentially partitioned with hexane, dichloromethane, ethyl acetate, and methanol. The latter was found to be the most active and was subjected to further purification. Methodical reversed-phase chromatographic purification led to the isolation of the alkaloid aaptamine. The structure and complete ^1^H and ^13^C NMR spectral assignments for the compound were determined based on extensive 1D and 2D NMR studies (see Supporting Information for NMR spectra, Figures S1–S5).

**Figure 1:**
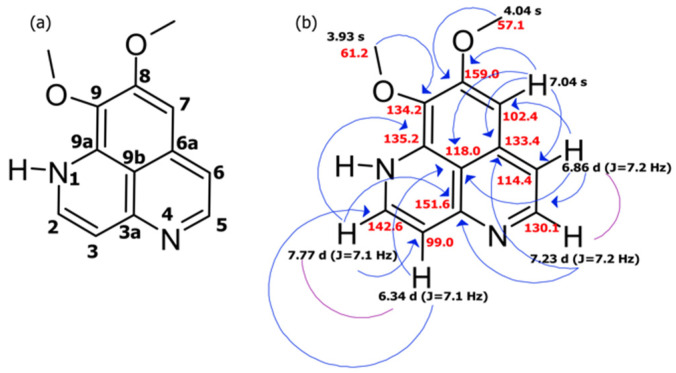
The purified alkaloid, aaptamine. (**a**) Structure of aaptamine. (**b**) HMBC (blue) and COSY (purple) correlations.

Aaptamine, the major component of the aqueous MeOH fraction, was obtained as bright yellow orange crystals. The HRESIMS in the positive mode displayed a protonated molecular ion [M+H] ^+^ peak at *m*/*z* 229.0972 (Supporting Information, Figure S6), indicative of the molecular formula C_13_H_12_N_2_O_2_, with six degrees of unsaturation. The ^1^H and ^13^C NMR signals were assigned to five aromatic methines (~δ_H_ 6–7 ppm) and two O-methyl groups (~δ_H_ 3–4 ppm). Detailed examination of COSY, HSQC, and HMBC data (Table 1) enabled the complete structural determination. The COSY data and the coupling constants for the five aromatic protons H-2 (δ_H_ 7.77, d, *J* = 7.1 Hz), H-3 (δ_H_ 6.34, d, *J* = 7.1 Hz), H-5 (δ_H_ 7.23, d, *J* = 7.2 Hz), H-6 (δ_H_ 6.86, d, *J* = 7.2 Hz), and H-7 (δ_H_ 7.04, s) (Table 1) denoted the presence of three fused benzene rings. HMBC correlations from a methoxy group (δ_H_ 4.04) to the quaternary carbon C-8 (δc 159.0) and the other methoxy group (δ_H_ 3.93) to C-9 (δc 134.2) justified the assigned location of these two methoxy groups. A combination of COSY and HMBC correlation data (Figure 1) established a planar polycyclic aromatic structure. The tabulated spectroscopic data matched the literature values of aaptamine, as previously described from the Okinawan marine sponge *Aaptos aaptos* [1].

### 2.2. Antiproliferative Activity of Aaptamine

**Figure 2:**
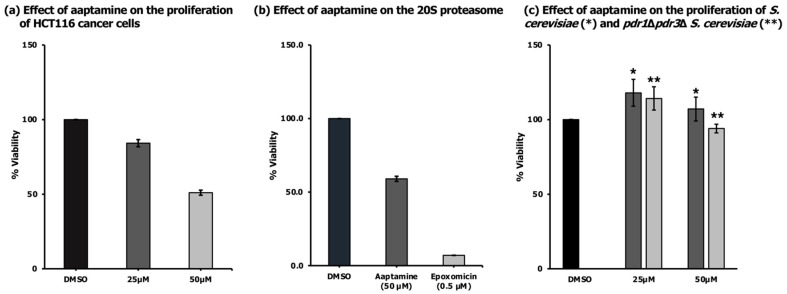
Activity of aaptamine on cancer cell viability, a potentially functionally related target (proteasome), and yeast cell viability. (**a**) Effect of aaptamine on the proliferation of HCT116 cancer cells. HCT116 cells were incubated for 48 h in the presence of different concentrations of aaptamine and the effects were compared to a solvent control (DMSO). (**b**) Effect of aaptamine on the purified 20S proteasome from human erythrocyte using Suc-LLVY-AMC fluorogenic peptide as the substrate. The proteasome and aaptamine (50 μM) were incubated for 10 min to allow inhibitor/enzyme interactions. Epoxomicin (0.5 μM) served as the positive control. (**c**) Effect of aaptamine on the proliferation of *S. cerevisiae* (*) and *pdr1*Δ*pdr3*Δ *S. cerevisiae* (**). The strain was incubated for 24 h in the presence of different concentrations of aaptamine and the effects were compared to the solvent control (DMSO). Data are presented as mean ± SD (n = 3), relative to 0.5% DMSO treatment + vehicle.

Aaptamine was tested against HCT116 colon cancer cells and cell viability was assessed after 48 h using an MTT (3-(4,5-dimethyl dimethylthiazol-2-yl)-2,5-diphenyltetrazolium bromide) assay. The compound reduced the viability of HCT116 by 50% at the highest concentration tested (50 µM, Figure 2a). The results agreed with the findings of Kobayashi et al. that aaptamine at concentrations of 20–50 µM increased the expression of the cyclin-dependent kinase inhibitor p21, a protein known to act as a negative regulator of cell cycle progression, thus preventing cell proliferation [15]. The Hamann group suggested that aaptamine’s antiviral and cytotoxic mechanism of action is due to DNA intercalation [16]. To alternatively explain the activity of aaptamine, we tested the compound for its ability to inhibit the chymotrypsin-like activity of a purified 20S proteasome from human erythrocytes. Proteasome inhibitors are validated anticancer agents [17,18]. Aaptamine was able to inhibit the enzyme activity by 41% at 50 µM (Figure 2b). Tsukamoto et al. reported that the proteasome inhibitory activity (IC_50_ value of 19 µM) of aaptamine did not correlate with its cytotoxicity against HeLa cells (IC_50_ value of 66 µM) [5]. In our study, aaptamine had similar IC_50_ values for cancer cell growth inhibition and proteasome inhibition in vitro (~50 µM).

It has been previously reported that aaptamine has moderate antifungal activity (MIC 110 µM) against *Candida tropicalis* [3]. We tested the compound against *Saccharomyces cerevisiae* (*S. cerevisiae*) and did not observe growth inhibitory activity against this strain up to the highest concentration tested (50 µM, Figure 2c). At the same concentrations, aaptamine was also inactive against a pleiotropic drug resistance (PDR)-attenuated *S. cerevisiae*, which was depleted of two homologous proteins, PDR1 and PDR3 (Figure 2c). The lack of activity of aaptamine against both wild-type and drug pump mutant *S. cerevisiae* suggests specific targets in mammalian cells or poor penetration to the yeast cell membrane.

### 2.3. GPCR Analysis

**Figure 3:**
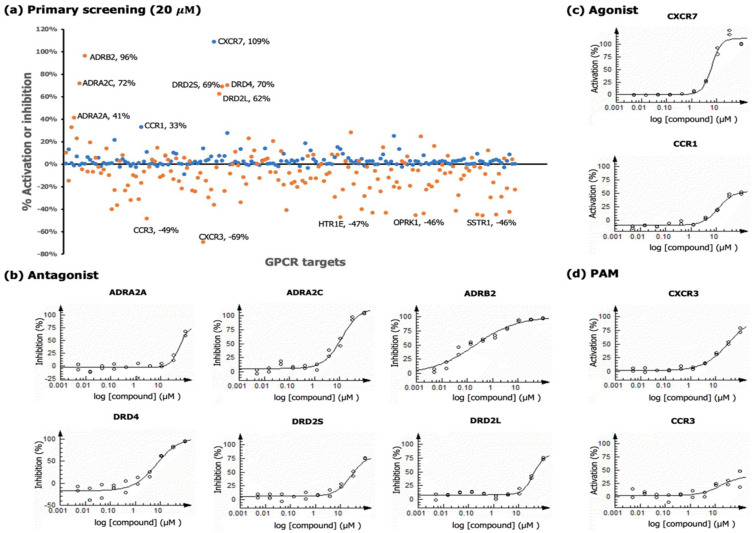
GPCR profiling of aaptamine using cell-based PathHunter β-arrestin assays under agonist and antagonist modes at 20 µM final concentration. Compound % activation or inhibition is calculated relative to basal and maximal activity values obtained through treatment of corresponding target ligand (100% at EC_80_). (**a**) Primary screening results shown in scatterplot against a panel of 168 GPCRs. The hits identified with >30% activation and >35% inhibition are labeled. (**b**) Dose-response curves of aaptamine in antagonist mode against confirmed hits: ADRA2A (IC_50_ 56.1 µM), ADRA2C (IC_50_ 11.9 µM), ADRB2 (IC_50_ 0.20 µM), DRD4 (IC_50_ 6.9 µM), DRD2L (IC_50_ 38.7 µM), and DRD2S (IC_50_ 25.7 µM). Yohimbine HCl (IC_50_ 0.091 µM), UK 14,304 (IC_50_ 1.5 × 10^−3^ µM), ICI 118,551 (IC_50_ 3.9 × 10^−4^ µM), Risperidone (IC_50_ 0.022 µM), Risperidone (IC_50_ 1.8 × 10^−3^ µM), and Risperidone (IC_50_ 0.022 µM) were the ligands used as positive controls for ADRA2A, ADRA2C, ADRB2, DRD4, DRD2L, and DRD2S, respectively. (**c**) Dose-response curves of aaptamine in the agonist mode against confirmed hits: CXCR7 (EC_50_ 6.2 µM) and CCR1 (EC_50_ 11.8 µM). CXCL12 (EC_50_ 9.6 × 10^−3^ µM) and CCL13 (EC_50_ 0.026 µM) were the ligands used as positive controls, respectively. (**d**) Dose-response curves of aaptamine in positive allosteric modulation (PAM) against CCR3 (EC_50_ 16.2 µM) and CXCR3 (EC_50_ 31.8 µM). The control agonist was added at EC_20_ to determine superactivation in the presence of low levels of agonists. CCL3 (EC_50_ 3.0 × 10^−3^ µM) and CXCL11 (EC_50_ 6.7 × 10^−3^ µM) were the ligands used as positive controls, respectively. Data are presented as mean ± SD (n = 2).

Aaptamine was profiled against a panel of 168 GPCR targets (agonist and antagonist modes) using cell-based functional assays at 20 μM, which was below the inhibitory concentration in our cell viability assays in HCT116 cancer cells. The screen was carried out using PathHunter β-arrestin assay technology (DiscoverX, CA). According to the manufacturer’s manual, a percentage activity of >30% and percentage inhibition of >35% are potentially significant. In the primary screen (Figure 3a), a total of 13 hits were observed: (a) two hits for agonist activity (CCR1 and CXCR7), (b) six hits for antagonistic activity (ADRA2A, ADRA2C, ADRB2, DRD2L, DRD2S, and DRD4), and (c) five hits in the antagonist mode as potentiators of agonist activity, when the control agonist was present at EC_80_ (CCR3, CXCR3, HTR1E, OPRK1, and SSTR1).

The hits were then subjected to dose-response analyses to obtain the IC_50_ (antagonist) and EC_50_ (agonist) values of aaptamine (Figure 3b,c). In the positive allosteric modulator (PAM) mode, the control agonist was added at EC_20_ to determine superactivation in the presence of a low level of agonist (potentiators), to increase the dynamic range (Figure 3d). Three potentiator hits in the primary screen did not validate under PAM conditions: 5-hydroxytryptamine receptor 1E (HTR1E), opioid receptor kappa 1 (OPRK1), and somatostatin receptor 1 (SSTR1). The corresponding dose-response curves are shown in the Supporting Information (Figure S7). The opioid receptor (OPR) consists of at least three distinct receptors: mu (μ), kappa (κ), and delta (δ). Johnson et al. reported that aaptamine possessed agonistic activity to opioid receptor delta 1 (OPRD1) via the calcium immobilization pathway with an EC_50_ value of 5.1 μM [6]. In our study, aaptamine did not demonstrate significant agonistic activity in the β-arrestin assay, potentially indicating signaling bias, which warrants further investigation. All other hits were validated, the most noteworthy being ADRB2, DRD4, and CXCR7 with an EC_50_/IC_50_ of <10 μM (Table 2).

#### 2.3.1. Chemokine Receptors

Chemokine receptors, which belong to the class A rhodopsin-like family of GPCRs, are considered one of the superfamilies of GPCRs that regulate immune cell behavior; they promote chemotaxis, cell adhesion, and mediator release [19]. The role of chemokines is extended to other pathological and physiological conditions including angiogenesis, hematopoiesis, atherosclerosis, and cancer [20]. The chemokine receptors can be classified into four subfamilies (CXC, CC, CX3C, and C) based on their conserved cysteine residues from the N-terminal. This subfamily of GPCRs is difficult for drug development, with only three Food and Drug Administration-approved compounds on the market (Selzentry^®^ targeting CCR5 in HIV/AIDS treatment, and Mozobil^®^ and Poteligeo^®^ targeting CXCR4 for hematopoietic stem cell mobilization and non-Hodgkin lymphoma, respectively) [7,21]. In this study, arrestin signaling through four chemokine receptors (CCR1, CCR3, CXCR3, and CXCR7) was significantly activated by aaptamine alone or in the presence of an agonist. Aaptamine displayed agonistic activity against CXCR7 (122.6% activation; EC_50_ 6.2 µM) and CCR1 (50.2% activation; EC_50_ 11.8 µM). Moreover, aaptamine appeared to act as a weak positive allosteric modulator for CXCR3 (74.6% activation; EC_50_ 31.8 µM) and CCR3 (33% activation; EC_50_ 16.2 µM) at an EC_20_ agonist concentration.

The CC-type chemokine receptor 1 (CCR1) is one of the frequent targets of drug development as reflected by the distribution of patents for small molecule inhibitors of chemokine receptors [22]. CCR1 is widely expressed by neutrophils, T cells, B lymphocytes, natural killer cells, monocytes, and CD34^+^ bone marrow cells. Diseases associated with CCR1 inactivation include Behcet Syndrome (a widespread inflammation of blood vessels) [23] and chromoblastomycosis (a chronic fungal infection) [24]. Thus, activation of this receptor is important. The remaining three chemokine receptors activated by aaptamine are known as progenitors of cancer development and metastasis. CC-type chemokine receptor 3 is linked with the occurrence of belligerent disease with extended local spreading and a higher risk of biochemical recurrence [25]. Examples of these are prostate cancer, thyroid, colon cancer, and glioblastoma [26]. CXC-type chemokine receptor 3 (CXCR3) is reported to be an important biomarker for the invasive subtype of kidney cancer, renal cell carcinoma [27]. On the other hand, CXC-type chemokine receptor 7 (CXCR7), activated by aaptamine, is widely expressed in different organs including the bone marrow, liver, heart, kidney, thymus, stomach, lymph nodes, pituitary gland, and brain [28]. The stromal cell-derived factor (SDF-1), also known as CXCL12, is the endogenous ligand of CXCR7, and is highly induced under certain pathological conditions including ischemia, inflammation, hypoxia, cancer, and autoimmune diseases [28,29]. As a family of cytokines, the CXC chemokines are pleiotropic in their ability to regulate tumor-associated angiogenesis, heart diseases [30], and cancer cell metastases [31]. FC313, TC14012, brintonamide D, and amantamide are examples of peptide-based modulators of CXCR7. FC313 is a pentapeptide ligand that recruits β-arrestin with an EC_50_ of 0.49 µM [32]. TC14012 is a cyclic peptide with an EC_50_ value of 3.50 nM against CXCR7 [33]. Cyclic peptide was observed to reduce fibrosis and alveolar repair in mice with lung injuries [34]. Linear depsipeptide brintonamide D targets several GPCRs, including CXCR7 (agonism); however, only antagonism of chemokine receptor type 10 (CCR10) was conclusively linked to the inhibition of (CCL27-induced) proliferation and the migration of breast cancer cells [35]. The linear peptide amantamide was identified as a selective agonist of CXCR7 (EC_50_ of 2.5 µM) [36]. It is believed that there are two possible mechanisms of action for the agonists: (a) downregulation of CXCR4 (tumor growth promoter), as CXCR7 is upregulated [37], and (b) desensitization or loss of function of CXCR7 due to prolonged exposure to an agonist [38]. Overall, pharmacological activation of CCR1, CXCR3, and CXCR7 may be useful for the development of novel therapies for a wide range of human diseases.

#### 2.3.2. Adrenergic Receptors

Adrenergic receptors (also known as adrenoreceptors, ADRs) belong to the guanine nucleotide-binding G protein-coupled receptor superfamily. There are two classes of ADRs that have been identified: ADRA (α-adrenoreceptor) and ADRB (β-adrenoreceptor). The subfamily of ADRA1 consists of three highly homologous subtypes: ADRA1A, ADRA1B, and ADRA1D [39]. The ADRA2 subfamily comprises three subtypes: ADRA2A, ADRA2B, and ADRA2C. Some species other than humans express the fourth homologous type, ADRA2D. In the ADRB family, there are three receptor subtypes: ADRB1 is found abundantly in the heart, ADRB2 is expressed extensively throughout the body, and ADRB3 is selectively expressed in the white and brown adipose tissue [39]. In this study, aaptamine displayed antagonistic activity against ADRA2A (63.4% inhibition; IC_50_ 56.1 µM), ADRA2C (103.9% inhibition; IC_50_ 11.9 µM), and ADRB2 (96.5% inhibition; IC_50_ 0.20 µM). 

The β2-adrenergic receptor (ADRB2) is the primary target of catecholamine epinephrine during the stress response. ADRB2 activation regulates various biological functions, including the heart rate, blood pressure, or respiration, and it may modulate the vasodilation of the microcirculation in normal coronary arteries [40]. Genetic polymorphisms in ADRB2 have been implicated in abnormal function of the receptor, which leads to increased vasodilation and susceptibility to myocardial infarction and coronary artery disease [40]. Antagonists of this type are called beta blockers. The β-adrenergic antagonistic activity of aaptamine has not been reported so far; hence, this is the first report for the compound.

α2-adrenoreceptor (ADRA2) is widely distributed throughout the body. Like ADRB2, α2-adrenoreceptors are expressed in the heart [41]. The widely characterized action of the ADRA2 is the inhibition of neurotransmitter release from peripheral and central neurons [42]. The activation of ADRA2 mediates actions such as smooth muscle contraction, platelet aggregation, and inhibition of insulin secretion [42]. Moreover, overexpression of ADRA2A suppresses insulin release, which is associated with the risk of type 2 diabetes [43], while ADRA2C overexpression is reported to have a behavioral despair and performance impairments [42]. Ohizumi et al. first reported the antagonistic activity of aaptamine against the α-adrenoreceptor using vascular smooth muscle of male albino rabbits, based on displacement analysis, finding the effective concentration at 3 × 10^−5^ M [14]. Their paper also discussed structure–activity relationship (SAR) studies of aaptamine and concluded that the aromatic property of the tricyclic ring system of aaptamine may play an important role in the development of α-adrenoreceptor blocking activity. However, the paper did not specify the type of α-adrenoreceptor. Since then, no reports have been published regarding the adrenergic activity of the compound. Thus, the antagonistic activity of aaptamine against ADRA2A and ADRA2C is a good addition to the GPCR signaling activities of the compound to the α-adrenoreceptor.

#### 2.3.3. Dopamine Receptors

Dopamine receptors (DR) are immensely distributed within the brain and play a critical role in modulating motor functions, motivation and drive, and cognition [44]. There are different dopamine receptor subtypes, pharmacologically grouped as DRD1- (DRD1 and DRD5) and DRD2-like (DRD2S, DRD2L, DRD3, and DRD4) [44]. DRD2-like receptors are the main targets of antipsychotics [45]. The DRD2 has two isoforms: D2S and D2L, which differ because of a 29-amino-acid insertion in the third loop on D2L [46]. DRD2 plays an important role in controlling emotion, cognitive, and sensory functions’ alterations in schizophrenia [47]. The D2S receptor is always present whenever schizophrenia treatment resistance is discussed [47]. On the other hand, the dopamine D4 receptor (DRD4) is a receptor that controls creativity. DRD4 reviews have reported that the 7R allele of the receptor is a risk allele for attention-deficit hyperactivity disorder (ADHD) [48], a disorder characterized by high dysfunctional impulsivity. The 7R allele, as a risk allele for executive dysfunction, flexibility, and cognitive shifting, can be correlated with low creativity [49]. In addition to ADHD, studies have shown that drugs target DRD4 for the treatment of schizophrenia, Parkinson’s disease, depression, and psychostimulant addiction [50,51]. In this study, aaptamine displayed an antagonistic activity on the dopaminergic system’s signals through DRD2-like subtypes: DRD2Long (L) (73.7% inhibition; IC_50_ 38.7 µM), DRD2Short (S) (74.4% inhibition; IC_50_ 25.7 µM), and particularly, DRD4 (94.5% inhibition; IC_50_ 6.9 µM). The dopamine-antagonistic activity of aaptamine holds promise for the treatment of mental problems.

## 3. Materials and Methods

### 3.1. General Experimental Procedures

The ^1^H, ^13^C, and 2D NMR spectra were recorded on a Bruker AVANCE II 600 MHz spectrometer (Billerica, MA, USA). The chemical shifts of the ^1^H NMR spectra were referenced using the residual solvent signal of Methanol-*d*_4_ at 3.31 ppm and at the center of the Methanol-*d*_4_ septet at 49.15 ppm in the ^13^C NMR spectra. HRMS data was collected on a High-Resolution LC Q-Exactive orbitrap mass spectrometer (ThermoSci, MA, USA).

### 3.2. Collection and Sample Preparation

The marine sponge sample was collected off the coast of Surigao City by hand scuba at a depth of 5–10 m by local sea divers. The sample was then stored in sterile containers and transported to the Natural Products and Bioorganic Research Laboratory of the Department of Chemistry and the Premier Research Institute of Science and Mathematics (PRISM) at Mindanao State University—Iligan Institute of Technology (MSU-IIT), Iligan City, Philippines. Taxonomic identification of the marine sample was done by Prof. Ephrime Metillo of the Department of Biological Sciences of MSU-IIT.

### 3.3. Extraction and Isolation

The freeze-dried and pulverized marine sponge sample was extracted with 50% methanol and 50% ethyl acetate at room temperature for three days. The mixture was then filtered, concentrated *in vacuo* using a rotary evaporator, and weighed to give the crude extract (2.4 g). The concentrated extract was dissolved in methanol (100 mL) to obtain the methanol fraction, while the remaining residue was dissolved in ethyl acetate to give the ethyl acetate fraction (80.3 mg; 3.35%). The methanol fraction was partitioned with hexane (100 mL) and the upper layer was aspirated to give the hexane fraction (770 mg; 28.5%), while water was added (50 mL) to the lower layer (methanol) to produce the aqueous methanol. Lastly, partitioning the aqueous fraction and dichloromethane (100 mL) gave the dichloromethane fraction (54.1 mg; 2.25%) and the aqueous methanol fraction (917 mg; 38.2%). Two portions of the aqueous methanol fraction were applied to a pre-packed HyperSep C_18_ 1 g column (ThermoSci, MA, USA) using milli-Q water with increasing amounts of methanol as the eluent. The fraction that eluted at 50% MeOH in H_2_O gave aaptamine. The aaptamine was comprised of bright yellow orange solid flakes (9.25 mg); UV (MeOH) λmax (log ε) 250 (3.75), 310 (3.19), 380 (3.01) nm; ^1^H and ^13^C NMR (for data, see Table 1); HRESIMS *m*/*z* 229.0972 Da [M + H]^+^ (calcd for C_13_H_12_N_2_O_2_, 228.08937 Da).

### 3.4. Bioassays 

#### 3.4.1. Antiproliferative Activity against Cancer Cells

Human colon carcinoma cells (HCT116) were cultured in Dulbecco’s modified Eagle’s medium (DMEM, Invitrogen, MA, USA), supplemented with 10% fetal bovine serum (FBS, Sigma-Aldrich, MO, USA) and 1% antibiotic-antimycotic (Invitrogen, MA, USA) humidified at 37 °C with 5% CO_2_. A 100-µL cell suspension containing 8000 cells was seeded in a 96-well microplate and incubated overnight at 37 °C and in a 5% CO_2_ atmosphere. After 24 h, 0.5 µL of aaptamine and DMSO (vehicle control) were added in triplicate and the cells were incubated for an additional 48 h. Detection was performed using a SpectraMax M5 spectrometer (Molecular Devices, CA, USA) at 570 nm and cell viability was calculated and expressed relative to the vehicle control.

#### 3.4.2. Proteasome Assay

The 20S proteasome assay kit from Enzo Life Sciences (NY, USA) is a fluorogenic, non-radioactive assay designed to measure the chymotrypsin-like protease activity of purified 20S proteasome from human erythrocyte. The Suc-LLVY-AMC fluorogenic peptide substrate supplied in the kit is a proteasome substrate used for measuring chymotrypsin-like peptidase activity. The detection of proteolytic activity is based on the release of free AMC fluorophore. Epoxomicin was used as a positive control. The assay was performed according to the manufacturer’s instructions. Briefly, a half-volume microtiter plate containing 25 μL assay buffer and 10 μL of 10 μg/mL 20S proteasome (0.1 μg/well as final concentration) were added with 5 μL of aaptamine (50 μM final concentration). The plate was incubated at room temperature for 10 min. After the incubation period, 10 μL of Suc-LLVY-AMC substrate was added to each well (75 μM final concentration). The fluorescence was then measured at an excitation of 360 nm and emission of 460 nm using a SpectraMax M5 plate reader (Molecular Device). The data were recorded every 2 min for 20 min. The assay was done in triplicate.

#### 3.4.3. Test for Activity against *Saccharomyces cerevisiae*

The yeast *S. cerevisiae* (BY4741) cell culture was prepared by inoculating the microorganism in a 250 mL Erlenmeyer flask containing 50 mL YPD + HEPES medium, followed by incubation at 30 °C for 24–48 h. The assay was performed in a 96-well format. Each well except for the blank contained 100 μL of cell suspension with a final cell density of 5 × 10^4^ cells/mL. The cell density was determined by measuring the absorbance at 660 nm using a SpectroMax M5 (Molecular Devices) spectrometer. From the 10-mM stock solution of aaptamine, 1 μL was added to each well except for the blank, which contained 100 μL of YPD medium. The absorbance was read after a 24 h incubation. A two-fold concentration was prepared from 10 mM aaptamine. Nystatin was used as the positive control and DMSO served as the negative control. Each treatment was done in triplicate.

#### 3.4.4. *S. Cerevisiae pdr1*Δ*pdr3*Δ Double Deletion Strain

The double deletion strain used in this study was obtained from Maya Schuldiner (Weizmann Institute of Science, Rehovot, Israel), as previously described and utilized [52]. The growth inhibition assay was performed similarly to the process described in Section 3.4.3.

#### 3.4.5. GPCR Profiling

Aaptamine (20 µM) was profiled in gpcrMAX panel biosensor assays (in agonist and antagonist modes) against a panel of 168 GPCRs. The assay was performed by DiscoverX Corporation (Fremont, CA) using PathHunter β-arrestin enzyme fragment complementation (EFC) technology, in a similar way to that described elsewhere [35,36]. The PathHunter cell lines were taken from freezer stocks according to standard procedures. A total of 20 µL of cells were seeded into white 384-well plates and incubated at 37 °C for the appropriate time prior to testing. 

For agonist determination, aaptamine was incubated with PathHunter cell lines to induce a response. To increase the concentration, intermediate dilution from 20 µM of aaptamine was performed to generate a 5X sample in an assay buffer. Then, 5 µL of 5X sample was added to the plate with PathHunter cells and incubated for 90 or 180 min at 37 °C or room temperature. The percentage activity of aaptamine was calculated following this formula: % activity = 100% × (mean RLU of test sample − mean RLU of vehicle control)/(mean MAX control ligand − mean RLU of vehicle control).

For antagonist determination, PathHunter cells were preincubated with aaptamine followed by the addition of an agonist. In this assay format, the standard agonist was added at an EC_80_ concentration. To the plate that contained the cells, 5 µL of the 5X sample was added and incubated for 30 min at 37 °C or room temperature. After the incubation period, 5 µL of the 6X EC_80_ agonist in an assay buffer was added to the cells and incubated for additional 90 or 180 min at 37 °C or room temperature. The percent inhibition of aaptamine was calculated following this formula: % inhibition = 100% × (1 − (mean RLU of test sample − mean RLU of vehicle control)/(mean RLU of EC_80_ control − mean RLU of vehicle control).

Hit validation was performed by subjecting the aaptamine to an agonist, antagonist, and positive allosteric modulation (PAM) secondary screen utilizing thirteen GPCR biosensor assays (Arrestin) against the following targets: ADRA2A, ADRA2C, ADRB2, CCR1, CCR3, CXCR3, CXCR7, DRD2L, DRD2S, DRD4, HTR1E, OPRK1, and SSTR1. The assays were performed at 10-point concentrations using three-fold serial dilutions in duplicate. For all the assay formats, the final assay vehicle concentration was 1%. 

For PAM assays, the cells were preincubated with the sample followed by agonist induction at the EC_20_ concentration. To the cells, 5 µL of 5X sample was added and incubated for 30 min at 37 °C or room temperature. Following the incubation period, 5 µL of the 6X EC_20_ agonist prepared in an assay buffer was added to the cells and incubated for 90 or 180 min at 37 °C or room temperature. The percent activity of aaptamine was calculated following this formula: % modulation = 100% × ((mean RLU of test sample − mean RLU of EC_20_ control)/(mean RLU of MAX control ligand − mean RLU of EC_20_ control)).

## 4. Conclusions

Chemical investigation of the Philippine marine sponge *Stylissa* sp. collected off Surigao City led to the isolation of the known alkaloid aaptamine. This is the first report for the compound in *Stylissa* sp. GPCR profiling revealed that aaptamine affects three large groups of GPCRs: chemokine receptors (immune cell migration and cancer), adrenoreceptors (cardiovascular, diabetes, and neural activity), and dopamine receptors (neural activity). Aaptamine is a promising starting point in the search for novel preventive and therapeutic strategies to treat several NCDs. The antagonistic activity of aaptamine upon α- and β-adrenergic receptors can be linked to treatment of heart failure. Moreover, the antagonistic activity upon α-adrenergic receptors can be correlated to its potential for the treatment of diabetes. Imbalanced dopamine production in the brain can lead to mental health problems. Mental health conditions are considered one of the major groups of NCDs. In this context, the blocking of a subset of dopamine receptors by aaptamine may be useful for the treatment of psychiatric conditions such as schizophrenia, bipolar disorder, depression, and psychostimulant addiction, which have been associated with an overactive dopamine system. The remarkable activity observed when activating CXCR7 can be useful for studying the biological role of CXCR7 and could also serve as a template for the development of therapeutic agents targeting CXCR7. Aaptamine showed submicromolar activity against ADRB2 with >50-fold selectivity over ADRA2C and 250-fold over ADRA2A, suggesting that the β-adrenoreceptor activity might be the most functionally relevant and promising for further exploration. Among the dopamine receptors, DRD4 was antagonized most potently with 3.7- to 5.6-fold selectivity.

To date, activation of CXCR7 and inhibition of ADRB2 and DRD4 signaling by aaptamine have not been reported. Hence, the findings gathered in this study are an important addition to the previously reported activities of aaptamine. However, the hits identified in this study need to be explored in the future, including signaling bias and selectivity profiles among similar receptors. 

## Figures and Tables

**Table 1:** NMR spectroscopic data (^1^H 600MHz, ^13^C 150 MHz, MeOD-*d_4_*) of aaptamine.

C/H No.	δ_c_, Type	δ_H_ (*J* in Hz)	COSY	HMBC
2	142.6 CH	7.77 d (*J* = 7.1)	H: 3	C: 3, 3a, 9a
3	99.0 CH	6.34 d (*J* = 7.1)	H: 2	C: 2, 9b
3a	151.6 C			
5	130.1 CH	7.23 d (*J* = 7.2)	H: 6	C: 3a, 6, 6a
6	114.4 CH	6.86 d (*J* = 7.2)	H: 5	C: 5, 7, 9b
6a	133.4 C			
7	102.4 CH	7.04 s		C: 6, 6a, 8, 9b
8	159.0 C			
9	134.2 C			
9a	135.2 C			
9b	118.0 C			
8-OMe	57.1 CH_3_	4.04 s		C: 8
9-OMe	61.2 CH_3_	3.93 s		C: 9

**Table 2:** Validated aaptamine activity as a positive allosteric modulator (PAM), antagonist, and agonist.

Assay Format	GPCR	Primary Screen	Dose-Response Analysis		
		Maximal Response, % Inhibition or Activation	Result Type	IC_50_ or EC_50_ Value, µM	Maximal Response, % Inhibition or Activation
Antagonist *^a^*	ADRB2	96	IC_50_	0.20	96.5
	DRD4	70	IC_50_	6.9	94.5
	ADRA2C	72	IC_50_	11.9	103.9
	DRD2S	69	IC_50_	25.7	74.4
	DRD2L	62	IC_50_	38.7	73.7
	ADRA2A	41	IC_50_	56.1	63.4
Agonist	CXCR7	109	EC_50_	6.2	122.57
	CCR1	33	EC_50_	11.8	50.2
PAM *^a^*	CCR3	49	EC_50_	16.2	33.0
	CXCR3	69	EC_50_	31.8	74.6

*^a^* The control agonist was added at EC_80_ in the primary screen and at EC_20_ in the dose-response analysis of the PAM mode. Dose-response curves for aaptamine against the ten GPCRs are shown in Figure 3b–d.

## Data Availability

Not applicable.

## Supplementary Materials

The following are available online, Figure S1. ^1^H NMR (600 MHz, MeOH-*d*_4_) spectrum of aaptamine, Figure S2. ^13^C NMR (150 MHz, MeOH-*d*_4_) spectrum of aaptamine, Figure S3. HSQC (600 MHz, MeOH-*d*_4_) spectrum of aaptamine, Figure S4. HMBC (600 MHz, MeOH-*d*_4_) spectrum of aaptamine, Figure S5. COSY (600 MHz, MeOH-*d*_4_) spectrum of aaptamine, Figure S6. Mass spectrum obtained by High Resolution Electrospray Ionization Mass Spectrometry (HRESIMS), Figure S7. Positive Allosteric Modulators (PAMs) assay data.

## Sample Availability

Samples of the compound are available from authors.
