# Supplementary material for: GPCR Pharmacological Profiling of Aaptamine from the Philippine Sponge Stylissa sp. Extends Its Therapeutic Potential for Noncommunicable Diseases
Source: Molecules. 2021 Sep 16;26(18):5618. doi: 10.3390/molecules26185618 (PMC8466755; doi:10.3390/molecules26185618)
Supplement: Supplementary file 1 [file molecules-26-05618-s001.zip › molecules-1359914-Supporting Information.pdf]

Supporting Information

GPCR Pharmacological Profiling of Aaptamine from the Philippine Sponge Stylissa sp. Extends its Therapeutic Potential for Noncommunicable Diseases

Harmie Luyao [1,2], Hendrik Luesch [2,*], and Mylene Uy, [1,3*]

[1]Department of Chemistry, Mindanao State University – Iligan Institute of Technology, Iligan City, [2]Philippines, 9200; harmie.luyao@g.msuiit.edu.ph
Department of Medicinal Chemistry and Center for Natural Products, Drug Discovery, and Development (CNPD3), University of Florida, Gainesville, Florida 32610, United States; luesch@cop.ufl.edu
[3]Premier Research Institute of Science and Mathematics (PRISM), Mindanao State University – Iligan Institute of Technology, Iligan City, Philippines; mylene.uy@g.msuiit.edu.ph

*Correspondence: mylene.uy@g.msuiit.edu.ph; luesch@cop.ufl.edu

Contents **Page Number**

Figure S1. ^{1}H NMR (600 MHz, MeOH-d_4) spectrum of aaptamine S2
Figure S2. ^{13}C NMR (150 MHz, MeOH-d_4) spectrum of aaptamine S3
Figure S3. HSQC (600 MHz, MeOH-d_4) spectrum of aaptamine S4
Figure S4. HMBC (600 MHz, MeOH-d_4) spectrum of aaptamine S5
Figure S5. COSY (600 MHz, MeOH-d_4) spectrum of aaptamine S6
Figure S6. Mass spectrum obtained by High Resolution Electrospray Ionization Mass Spectrometry (HRESIMS) S7
Figure S7. Positive Allosteric Modulators (PAMs) assay data S8

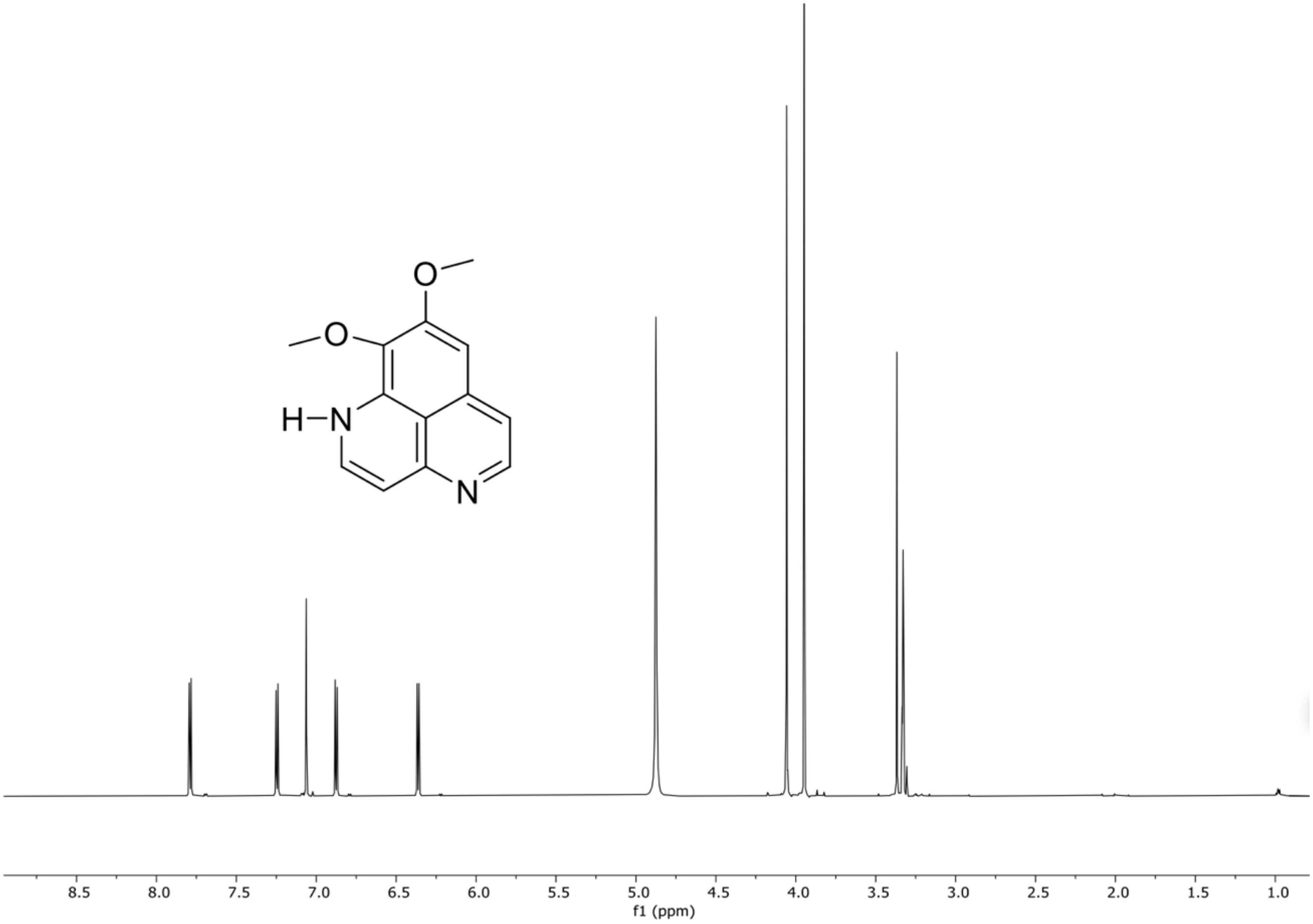

Figure S1. ^{1}H NMR (600 MHz, MeOH-d_4) spectrum of aaptamine.

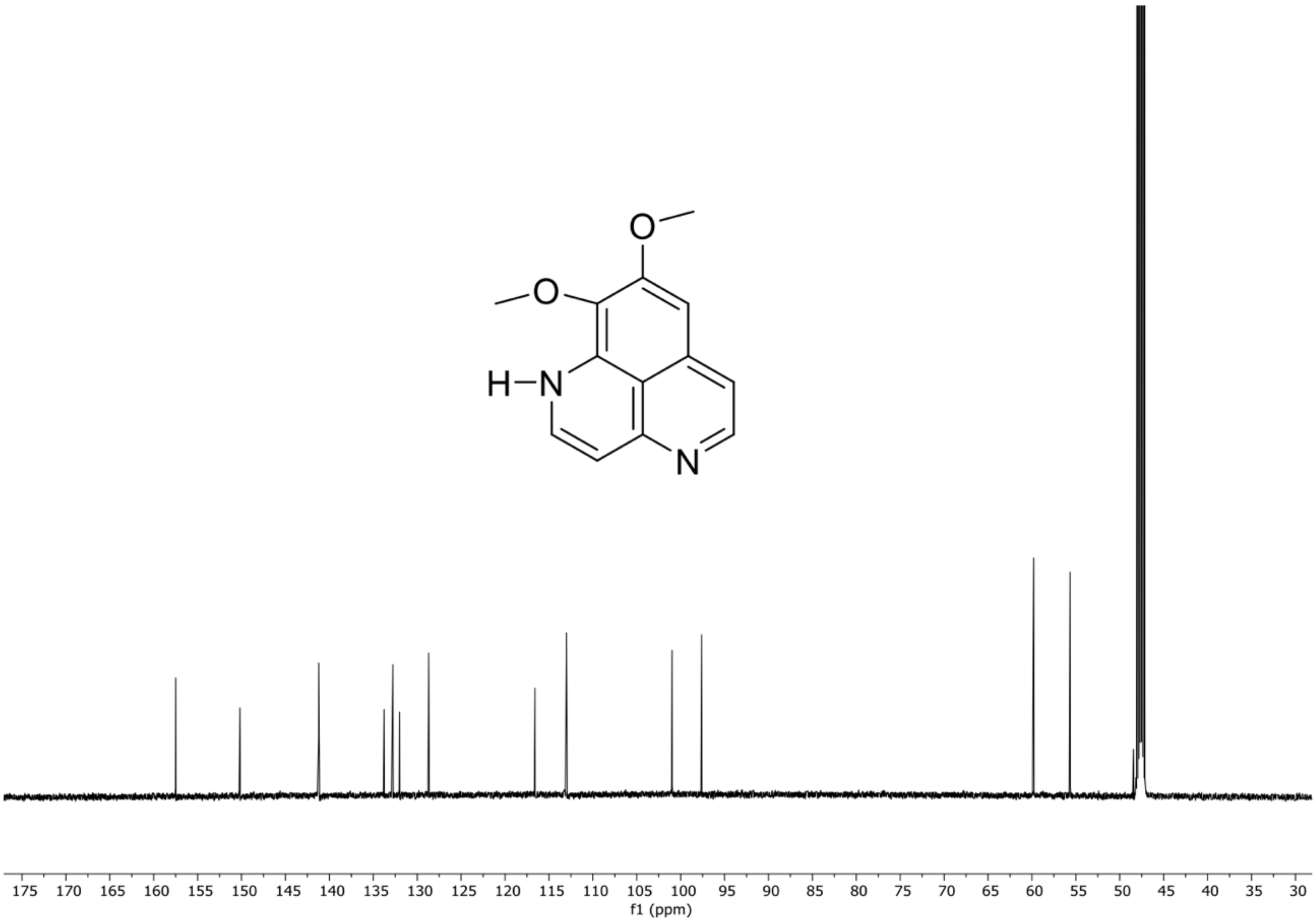

Figure S2. ^{13}C NMR (150 MHz, MeOH-d_4) spectrum of aaptamine.

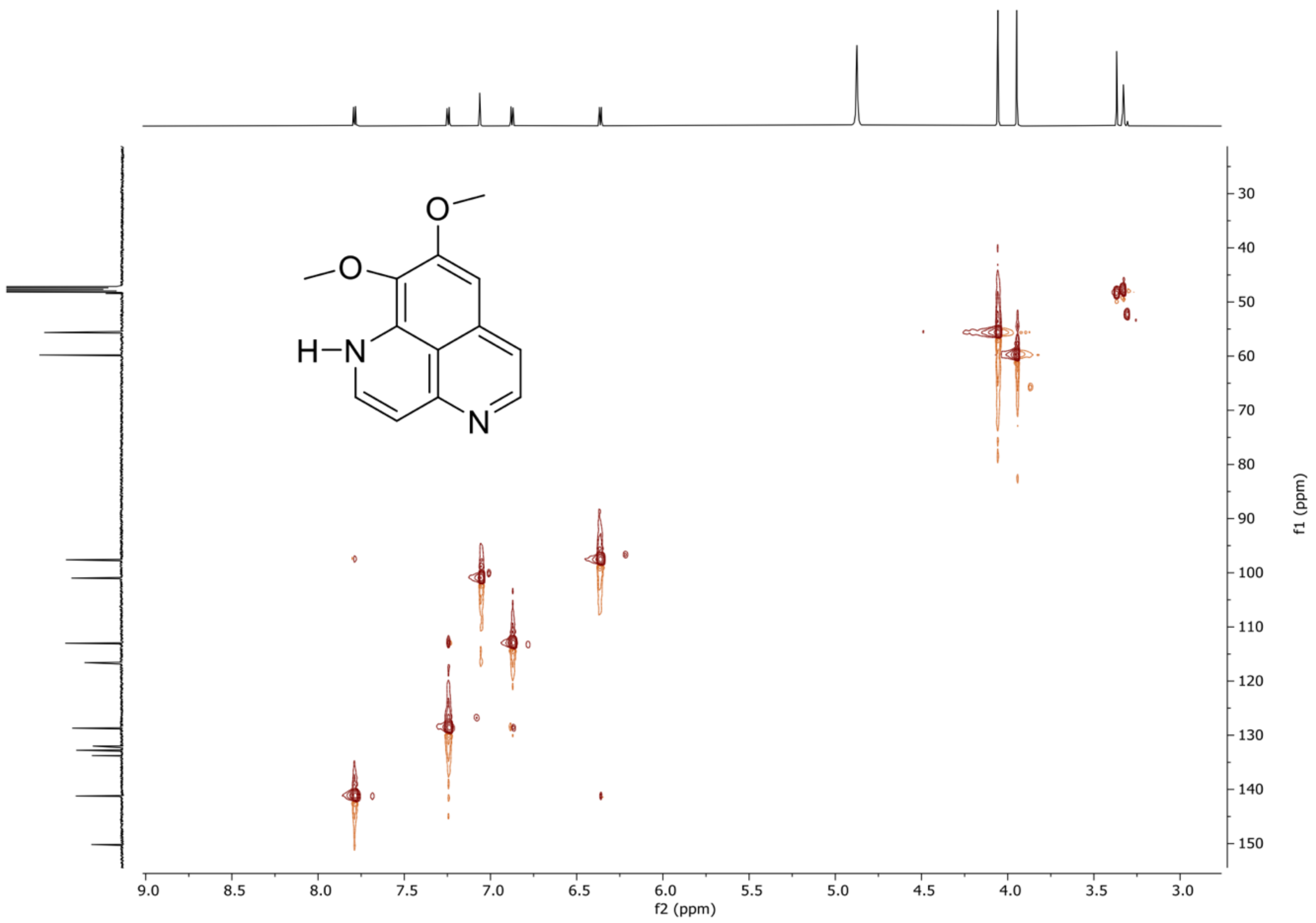

Figure S3. HSQC (600 MHz, MeOH-*d*$_4$) spectrum of aaptamine.

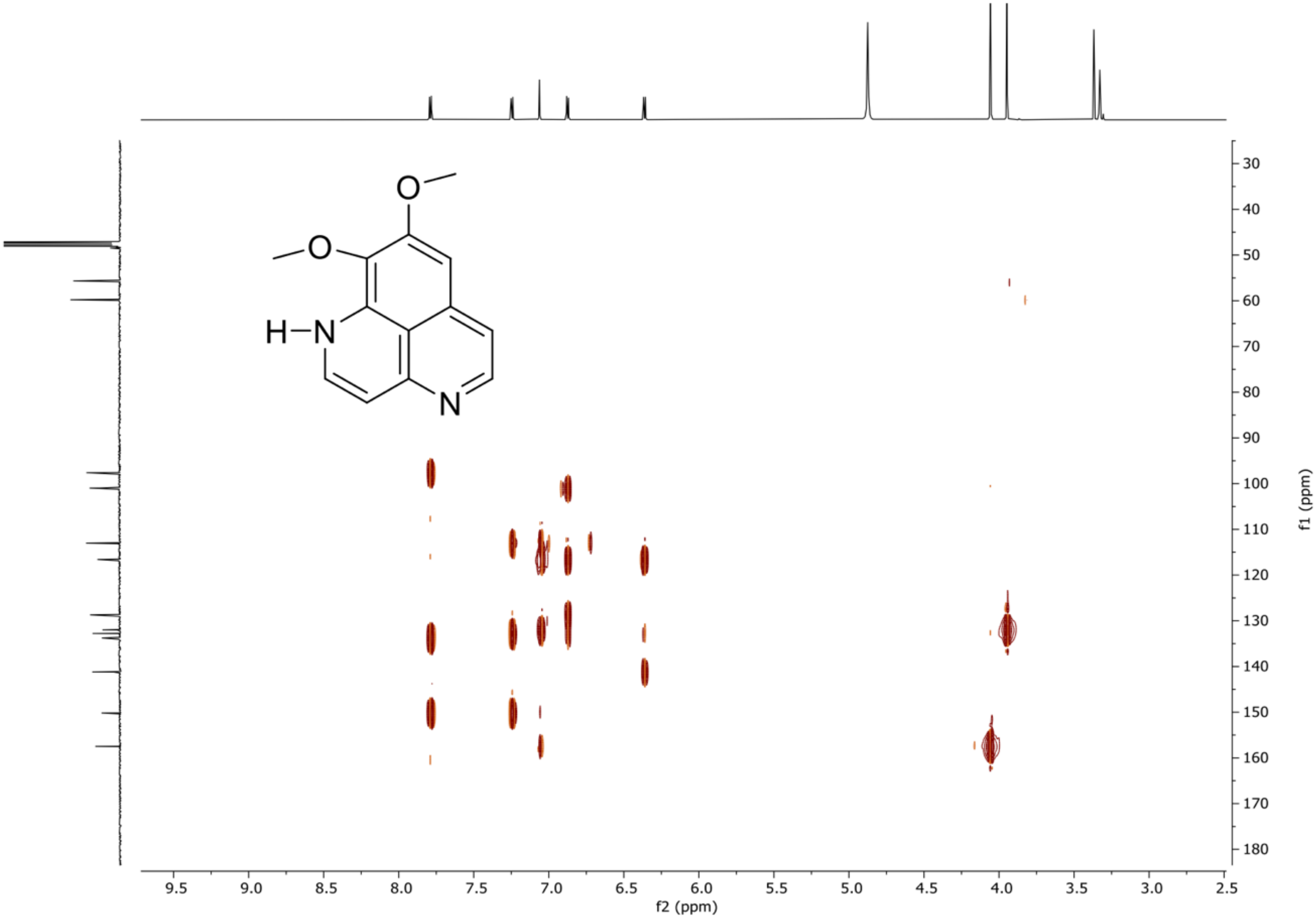

Figure S4. HMBC (600 MHz, MeOH-d_4) spectrum of aaptamine.

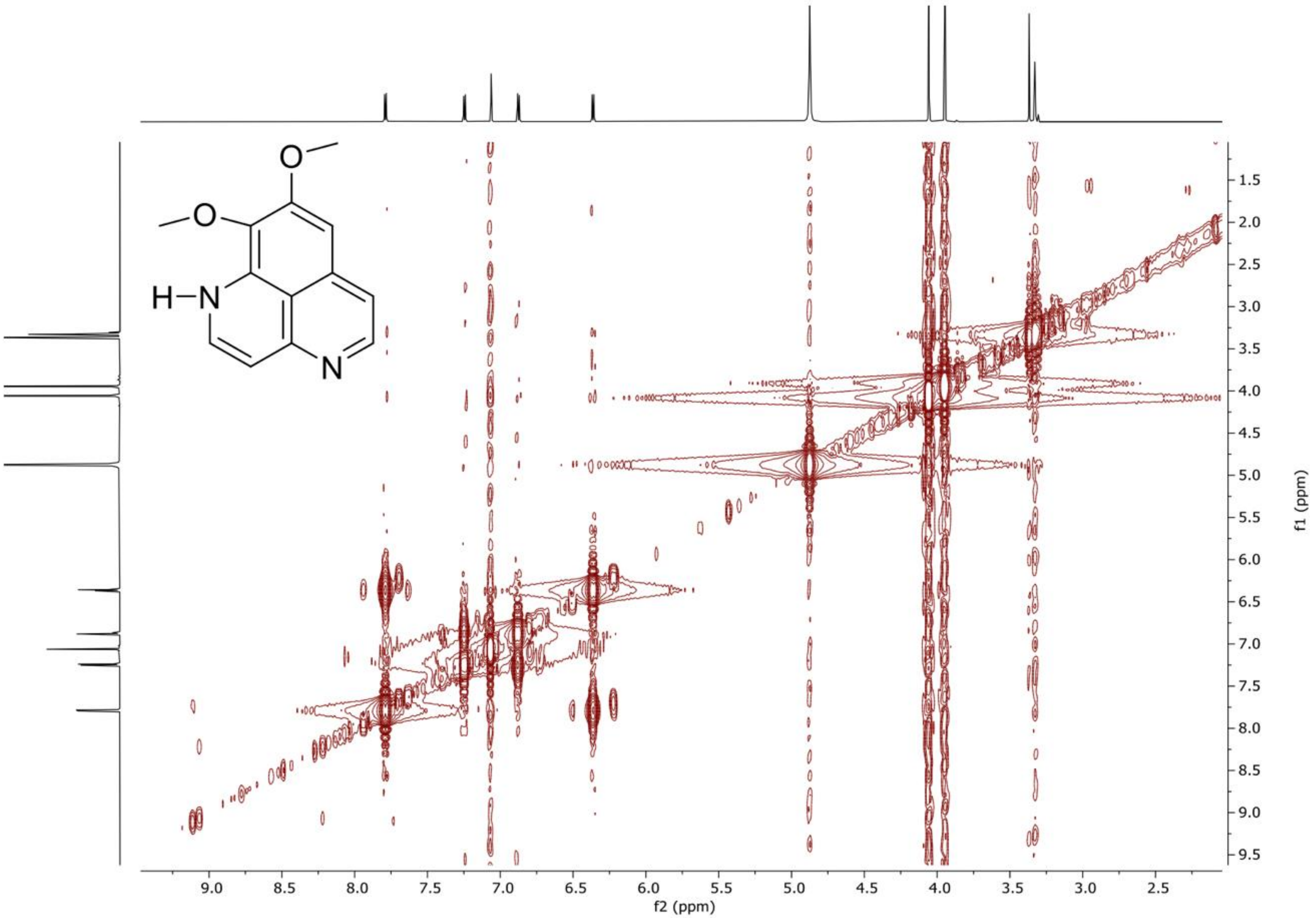

Figure S5. COSY (600 MHz, MeOH-d_4) spectrum of aaptamine.

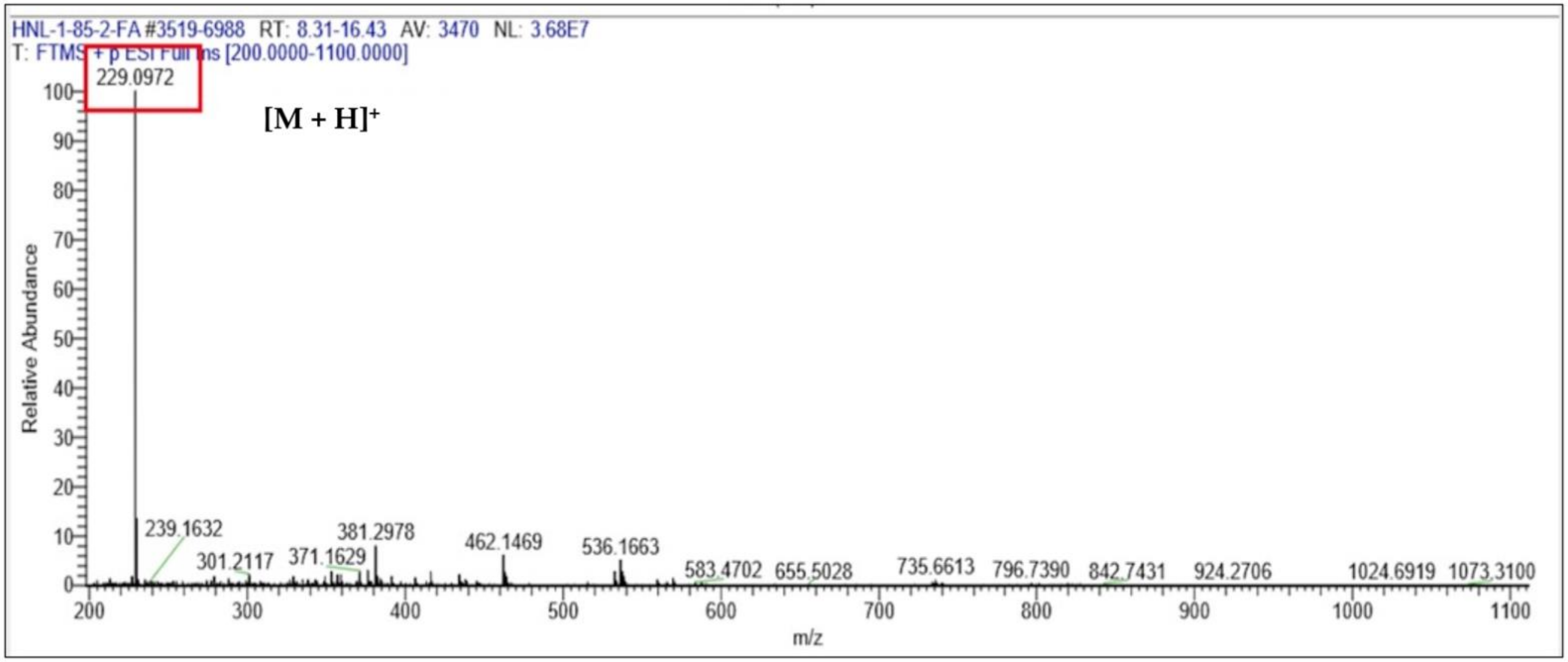

Figure S6. Mass spectrum obtained by High Resolution Electrospray Ionization Mass Spectrometry (HRESIMS).

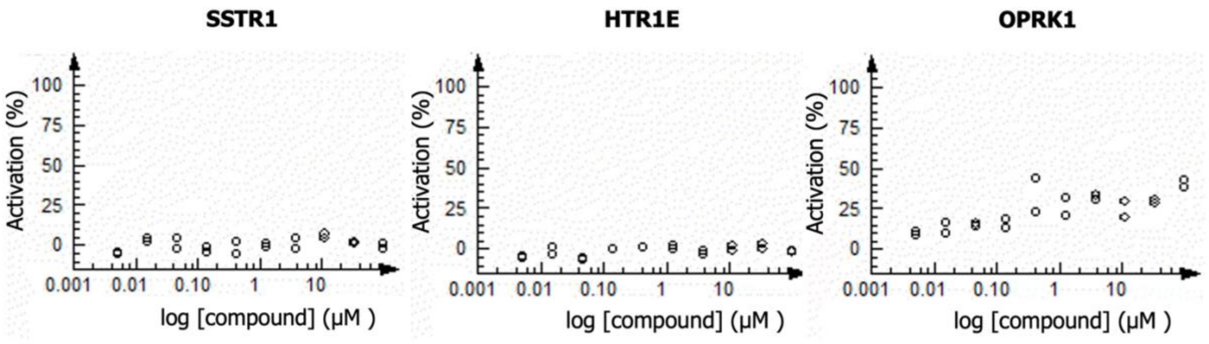

Figure S7. Positive Allosteric Modulators (PAMs) assay data. Dose-response curve of aaptamine against SSTR1, HTR1E, and OPRK1 observed in the secondary screening. The control agonist was added at EC_{20} to determine superactivation of the GPCR in the presence of the compound. The PAM mode showed that the hits identifed in the primary screen may be considered false positives. Somatostatin 28 (EC_{50} 4.0 x 10^{-3} μM), Serotonin/5-HT (EC_{50} 8.4 x 10^{-3} μM), and Dynorphin A (EC_{50} 3.4 x 10^{-2} μM) were the ligand used as positive control for SSTR1, HTR1E, and OPRK1, respectively. Data are presented as mean ± SD (n = 2).
